# Maternal depression is associated with injuries in children aged 2–4 years: the Pelotas 2004 Birth Cohort

**DOI:** 10.1136/injuryprev-2017-042641

**Published:** 2018-02-26

**Authors:** Raquel Siqueira Barcelos, Iná da Silva dos Santos, Alicia Matijasevich, Luciana Anselmi, Fernando Celso Barros

**Affiliations:** 1 Programa de Pós-graduação em Epidemiologia, Faculdade de Medicina, Universidade Federal de Pelotas, Pelotas, Brazil; 2 Departamento de Medicina Preventiva, Faculdade de Medicina, Universidade Federal de Sao Paulo, Sao Paulo, Brazil; 3 Programa de Pós-graduação em Saúde e Comportamento, Universidade Catolica de Pelotas, Pelotas, Rio Grande do Sul, Brazil

**Keywords:** cohort study, child, burn, fall, mental health

## Abstract

**Introduction:**

Injuries during childhood, which mostly consist of falls, burns, drowning, poisonings and car crashes, are among the main causes of death among children and young adults in several countries.

**Objectives:**

To investigate the association between maternal depression and the incidence of injuries during childhood.

**Methods:**

In 2004, children who were born in the municipality of Pelotas, Brazil, were enrolled in a population-based birth cohort, with evaluations at birth and at 3, 12, 24 and 48 months of age. Maternal depression during pregnancy was evaluated at the time of delivery. At 12 and 24 months post partum, the Edinburgh Postnatal Depression Scale (EPDS) was used. The injuries incidence rates at ages of 24–48 months and the crude and adjusted IRRs were calculated with 95% CI through Poisson’s regression.

**Results:**

A total of 3533 children were analysed. The incidence of injuries was higher among children whose mothers presented depressive symptoms during pregnancy and at 12 and 24 months compared with those whose mothers did not present any symptoms. In the adjusted analysis, the IRR among girls whose mothers presented depressive symptoms during pregnancy and EPDS ≥13 at 12 and 24 months was 1.31 (1.15–1.50); and, among boys, 1.18 (1.03–1.36).

**Interpretation:**

Maternal depression is associated with higher incidence of injuries between 24 and 48 months of age, in both sexes.

## Introduction

Injuries during childhood, which mostly comprise falls, burns, drowning, poisonings and car crashes,^1^ are a worldwide health problem, not only because of the morbidity that may arise, but also because such injuries are among the main causes of death among children and young adults in several countries.^2^ Childhood injuries are often interpreted as a work of chance, but most often predisposing factors play a role on its occurrence. A population-based study, conducted in the municipality of Pelotas, RS, Brazil, showed that the prevalence of falls, cuts and burns ranged from 5.2% for burns during the first year of life, among girls, to 77.7% for falls during the second year of life, among boys.^3^ In this same study, maternal factors such as younger age (<18 years), lower socioeconomic status and lower educational level were associated with higher incidence of injuries among children aged 0–4 years.^3^

Other factors, such as inadequate supervision, family stress, improper dwelling conditions and characteristics of the child’s personality, such as hyperactivity, aggressiveness, impulsivity and distraction, also facilitate the occurrence of injuries.^4^ As maternal depression is a common condition associated with several child health outcomes, some authors have suggested an association between maternal depression and incidence of injuries among children.^5 6^

One of the first studies evaluating the relationship of mental disorders in mothers and injuries in childhood was conducted in England in 1978. That study explored the association not specifically of depression but between maternal psychiatric disorders and injuries among individuals younger than 16 years of age. Among those whose mothers presented some kind of psychiatric disorder, the injury incidence rate per year was higher than among the controls.^7^ Other studies carried out in 2012 in Japan^8^ and the UK^9^ and more recently in 2017 in England^10^ have pointed out that maternal depression was related to childhood accidents, often increasing as the duration of depressive symptoms increased in the mother.

Thus, the specific objective of the present study was to investigate the association between maternal depression and the incidence of injuries among children from 2 to 4 years who belong to the Pelotas 2004 Birth Cohort, in Southern Brazil. We tested the hypothesis that children from depressed mothers had increased incidence of injuries from 2 to 4 years of age.

## Materials and methods

### Data collection

In 2004, a third Pelotas birth cohort study was started. In Pelotas, <1% of the deliveries occur outside hospitals.^11^ The Pelotas 2004 Birth Cohort included 4231 children who were born alive in all five maternity hospitals to mothers who lived in the urban area of the municipality. The refusal rate to participate in the study was 0.8%. So far, these children have been visited at the ages of 3, 12, 24, 48 and 72 months and 11 years, with follow-up rates of 95.7%, 94.3%, 93.5%, 92.0%, 90.2% and 86.6%, respectively. At the time of birth (perinatal study) and during the follow-up visits, information on a series of socioeconomic, demographic, behavioural and biological characteristics was collected both from the mother and from her child. This information was obtained by trained interviewers through the application of a structured and standardised questionnaire. Further details of the study are available in other publications.^12–14^

### Measurement

In the present study, information from the perinatal study and the follow-ups at 12, 24 and 48 months of age was used. Only children from single pregnancies were included in the analyses. The outcome (number of falls, cuts and burns that occurred between 24 and 48 months of age) was measured during the visit at 48 months. The section of the questionnaire about injuries began with the following questions: *Now I am going to ask you a few questions about injuries that<CHILD>  may have had.* Next, they were asked, *After he/she turned two years old, ‘did <CHILD> fall and got injured?’*, *‘did <CHILD> had a cut?’* and ‘*did <CHILD> got burned?’*, with the following answer options: ‘no’, ‘yes’ or ‘don’t know’. When the answer was ‘yes’, the person was then asked, *How many times?.* The child was considered to have suffered an injury if the mother or caretaker reported the occurrence of at least one of any of the three types of injuries.

The exposure of interest, maternal depression, was investigated in the perinatal study and in the follow-ups at 12 and 24 months of age. Maternal depression during pregnancy was investigated at the perinatal interview by means of the following question: *During pregnancy, did you have depression or suffer from nerves?*, with the following answer options: ‘no’, ‘yes, not treated’ or ‘yes, treated’. Because the number of mothers at the ‘treated group’ was low (n=117), the ‘treated’ and ‘not treated’ options were grouped into a single category for the present analysis. At 12 and 24 months of age, maternal depression was evaluated by means of the Edinburgh Postnatal Depression Scale (EPDS).^15^ For analysis purposes, the threshold cut-off ≥13 was used (sensitivity and specificity 59.6% and 88.3%, respectively).^16^ A composite variable was created from the responses obtained during the perinatal interview and from the EPDS results at 12 and 24 months of age, which was categorised into three groups: ‘never depressed’ (mothers who did not present depressive symptoms at any time); ‘depressed 1–2 times’ (mothers who reported depressive symptoms at one or two follow-ups) and ‘always depressed’ (mothers who presented depressive symptoms during pregnancy and at 12 and 24 months post partum).

The following variables collected in the perinatal study were used as potential confounding factors: mother’s age (<20, 21–30 or ≥31 years); Brazilian National Economic Index, divided into five quintiles and constructed from information on consumer goods and the head of the family’s educational level^17^; mother’s educational level (0–4, 5–8 or >8 years); mother lives with a partner (yes, no); planned pregnancy; maternal smoking during pregnancy (smoking at least one cigarette per day, every day, during any trimester of pregnancy); alcohol consumption during pregnancy (regular consumption at least once a week, regardless of the amount); child’s skin colour observed by the interviewer (white or black/brown/other); low birth weight (<2500 g) and prematurity (gestational age at birth <37 weeks). From the 24-month follow-up, the following variables were used: number of siblings (besides the index child) living in the household (none, 1–2 and ≥3); and type of family (nuclear—parents and children or extensive—including grandparents and/or other relatives).

### Analysis

**Figure 1:**
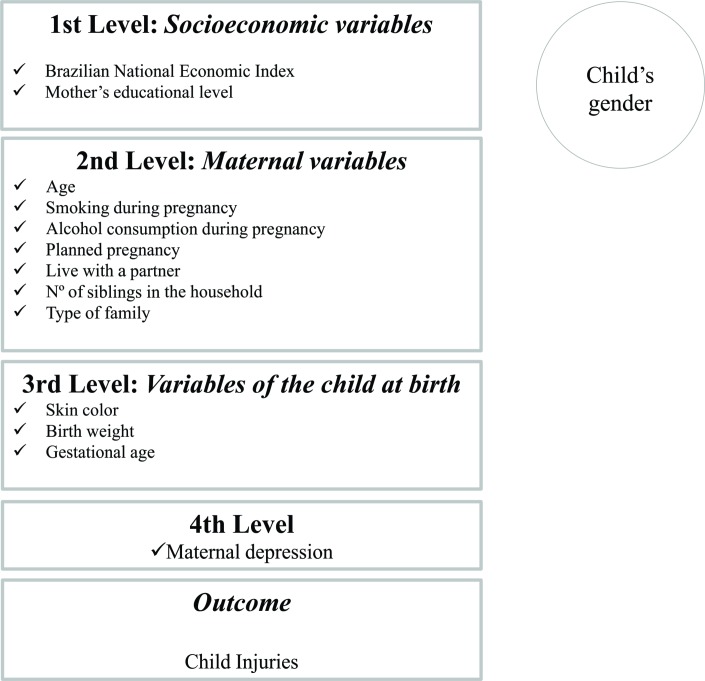
Model of analysis for the association between maternal depression and injuries in childhood.

Statistical analyses were performed using the Stata software, V.12.1 (StataCorp). The outcome variable was treated as a count (number of injuries suffered by the child between 24 and 48 months of age). First, the number of injuries was calculated, along with their distribution and the proportion of the children who suffered at least one injury between 24 and 48 months of age. Incidence rate according to the exposure of interest was then calculated. Crude and adjusted IRRs and 95% CIs were calculated by Poisson’s regression with robust variance. The variables were included in the analysis by levels, according to figure 1. At each level, the P value of the variables was verified, and those with P value<0.20 remained in the final model to control for possible confounding effect. Based on the literature, which is consistent in indicating higher incidences of injuries among boys, all analyses were planned to be stratified according to the child’s sex.^3 5 18^ Complementary analyses were run to assess the relationship between maternal depression and each of the types of injuries investigated.

A total of 94 children had died in the first four years and 338 were lost (n=234) or refused (n=51) to participate at the 48-month-follow-up. Therefore, 3533 children who had full information on maternal depression and injuries were entered in the analyses. Mean child age at the 48-month follow-up was 49.55 (SD ±1.77) months, ranging from 44.71 to 61.53 months. All mothers gave their free and informed consent in writing before the interview.

## Results

There was interaction between the child’s sex and the incidence of injuries (P=0.002). Table 1 shows the antenatal maternal characteristics and the characteristics of the children at birth. Almost one fifth of the mothers (18.6%) were adolescent mothers and 15% had low level of schooling (0–4 years of formal education). Only 35.2% of the mothers reported having planned their pregnancies. Unhealthy life habits during pregnancy, such as smoking and alcohol consumption, were reported by 26.8% and 3.3% of the mothers, respectively. Except for the variable ‘low birth weight’, for which the prevalence was higher among girls, all other characteristics were similar between the sexes.

**Table 1:** Maternal characteristics from pregnancy to 24 months post partum, children characteristics at birth and proportion of children who suffered injuries from 24 to 48 months of age: Pelotas 2004 Birth Cohort; Pelotas, RS, Brazil

Characteristics	Boys (n=1840)		Girls (n=1693)		Total (n=3533)	
	n	%	n	%	n	%
**Maternal characteristics**						
Brazilian National Economic Index (n=3533)						
Q1← poorest	355	19.3	315	18.6	670	19.0
Q2	333	18.1	348	20.6	681	19.3
Q3	364	19.8	359	21.2	723	20.5
Q4	400	21.7	332	19.6	732	20.7
Q5← richest	388	21.1	339	20.0	727	20.6
Age (years) (n=3531)						
<20	339	18.4	316	18.7	655	18.6
21–30	993	54.0	893	52.8	1886	53.4
≥31	508	27.1	482	28.5	990	28.0
Education level (years) (n=3499)						
0–4	271	14.8	252	15.1	523	15.0
5–8	743	40.7	692	41.4	1435	41.0
>8	814	44.5	727	43.5	1541	44.0
Live with a partner (yes) (n=3533)	1553	84.4	1434	84,7	2987	84.6
Number siblings in the household (24 months) (n=3533)						
None	878	47.7	720	42.5	1598	45.2
1–2	796	43.3	798	47.1	1594	45.1
≥3	166	9.0	175	10.3	341	9.7
Type of family (24 months) (n=3533)						
Nuclear	1393	75.7	1288	76.1	2681	75.9
Extensive	447	24.3	405	23.9	852	24.1
Planned pregnancy (yes) (n=3532)	677	36.8	567	33.5	1244	35.2
Smoking during pregnancy (yes) (n=3533)	489	26.6	457	27.0	946	26.8
Alcohol consumption during pregnancy (yes) (n=3533)	63	3.4	53	3.1	116	3.3
Maternal depression during pregnancy (yes) (n=3533)	450	24.5	409	24.2	859	24.3
Edinburgh Postnatal Depression Scale at 12 months≥13 (n=3533)	266	14.5	255	15.1	521	14.8
Edinburgh Postnatal Depression Scale at 24 months≥13 (n=3533)	277	15.1	280	16.5	557	15.8
**Child characteristics**						
Child’s skin colour (white) (n=3326)	1165	67.4	1074	67.3	2239	67.3
Low birth weight (<2500 g) (n=3532)	128	7.0	149	8.8	277	7.8
Prematurity (<37 weeks) (n=3529)	228	12.4	221	13.1	449	12.7
Injuries between 24 and 48 months (yes)						
Falls (n=3532)	1319	71.2	1145	67.6	2464	69.8
Cuts (n=3531)	590	32.1	485	28.6	1075	30.4
Burns (n=3532)	327	17.8	266	15.7	593	16.8

### Maternal depression

During pregnancy, 24.3% of the mothers reported having either treated or untreated depression. At 12 and 24 months after childbirth, 14.8% and 15.8% of the mothers presented EPDS ≥13 (table 1). Among the boys, 23.0% of the mothers had been depressed 1–2 times and 14.8% were always depressed, whereas 62.9% had never being depressed. Among the girls, 25.1% of the mothers had been depressed 1–2 times and 13.6% were always depressed, whereas 61.3% had never being depressed (data not shown).

### Injuries between 24 and 48 months

The proportion of children who suffered at least one injury was 77.8%, and this proportion was higher among boys (79.4%; 95% CI 77.5 to 81.2) than among girls (75.9%; 95% CI 73.9 to 77.9). The proportion of boys and girls who suffered falls was 71.2% and 67.6%; cuts 31.1% and 28.6%; and burns 17.8% and 15.7%, respectively (table 1). The median number of injuries among boys was higher among those from mothers who always presented symptoms of depression (median=4) (data not shown). For girls, the medians were similar among the daughters of mothers who had 1–2 episodes of depression and among those who were always depressed (median=3.5 for EPDS ≥13) (data not shown).

Table 2 shows the incidence rates of injuries according to episodes of maternal depression. Among the boys, the incidence of injuries was higher when mothers presented depressive symptoms at the three times evaluated (table 2). Among the girls, the incidence was higher when their mothers presented symptoms on at least one of the occasions evaluated (table 2).

**Table 2:** Incidence of injuries and their respective CIs (95% CI) according to episodes of maternal depression* (n=3533)

Maternal depression	Incidence of injuries (95% CI)		
	Boys	Girls	Total
Never depressed	7.79	6.22	7.05
	(7.62 to 7.95)	(6.07 to 6.37)	(6.94–7.16)
Depressed 1–2 times	7.96	8.26	8.11
	(7.69 to 8.23)	(8.0 to 8.4)	(7.92–8.31)
Always depressed	9.45	8.36	8.94
	(9.08 to 9.84)	(7.99 to 8.74)	(8.67–9.21)

*Edinburgh Postnatal Depression Scale ≥13.

Table 3 presents the crude and adjusted IRRs for injuries, according to maternal depressive symptoms. Both sexes presented higher incidence as the exposure to maternal depression increased, and girls presented higher risk than boys. In the adjusted analysis, the boys whose mothers were always depressed, the incidence of injuries was 18% (1.18; 95% CI 1.03 to 1.36) higher than what was observed among those whose mothers had never been depressed.

**Table 3:** IRRs of injuries among children aged 24–48 months, according to exposure to maternal depression during pregnancy and at 12 and 24 months post partum (EPDS ≥13): 2004 Pelotas Birth Cohort, RS, Brazil (n=3289)

Maternal depression	Injuries (95% CI)					
	Boys		Girls		Total	
	Crude	Adjusted*	Crude	Adjusted†	Crude	Adjusted‡
	P<0.001	P=0.03	P<0.001	P<0.001	P<0.001	P<0.001
Never depressed	1.0	1.0	1.0	1.0	1.0	1.0
Depression 1–2 times	1.02	1.00	1.33	1.31	1.15	1.16
	(0.98 to 1.06)	(0.88 to 1.14)	(1.28 to 1.38)	(1.15 to 1.50)	(1.12 to 1.18)	(1.06 to 1.28)
Always depressed	1.21	1.18	1.34	1.34	1.27	1.24
	(1.16 to 1.27)	(1.03 to 1.36)	(1.28 to 1.41)	(1.13 to 1.58)	(1.22 to 1.31)	(1.11 to 1.39)

Model adjusted for

*Brazilian National Economic Index, mother’s age, live with a partner and maternal smoking.

†Brazilian National Economic Index, mother’s education level, mother’s age, planned pregnancy, type of family.

‡Brazilian National Economic Index, mother’s education level, mother’s age, maternal smoking, planned pregnancy, child’s skin colour and birth weight.

Among the girls, in the adjusted analysis, the IRR values were quite similar between those whose mothers were depressed on 1–2 occasions or on all three occasions. The daughters whose mothers suffered 1–2 depressive episodes presented incidence that was 31% higher (1.31; 95% CI 1.15 to 1.50), and among those whose mothers were always depressed, the incidence was 34% higher (1.34; 95% CI 1.13 to 1.58) than among the daughters whose mothers were never depressed (table 3).

Analyses of association between maternal depression and each of the injuries separately showed that for falls the IRRs were similar to those observed for the composite variable (with the three types of injuries taken together) (online supplemental tables 2A and 3A). The incidence of cuts and burns showed the same direction of increased risk with increased exposure to maternal depression but with CIs including the value 1 (online supplemental tables 2B, 2C, 3B and 3C).

10.1136/injuryprev-2017-042641.supp1 Supplementary file 1

## Discussion

The results from the present study showed that maternal depression is associated with higher incidence of injuries among children of both sexes aged 2–4 years, even after adjustment for confounding factors. The risk was higher among girls.

This study is among the few that have investigated the association between maternal depression and injuries during childhood. A search in PubMed, Web of Science and PsycINFO databases, without publication year or language restrictions, found only four studies that specifically evaluated such association.^8–10 19^

A cohort study conducted in the USA with children under 6 years of age examined the relationship between depressive symptoms in mothers and injuries in children. The outcome included all lesions attended by a health professional, and the depressive symptoms of the mother were assessed by means of the Center for Epidemiologic Studies Depression Scale.^20^ In boys, the risk of reported injury increased by 4% for each one-point increase in maternal depressive symptoms. Among the girls, no association was found.^19^

A study conducted in Japan, in 2012, investigated the association between postpartum maternal depression and unintentional injuries among children aged 3–4 months. Children whose mothers had a EPDS score ≥9 were more likely to suffer some kind of injury than were children whose mothers were not depressed (OR 1.59; 95% CI 1.24 to 2.04).^8^

In the UK, in 2012, a case–control study using Nationwide Primary Healthcare Services database to investigate risk factors for the occurrence of the first medically recorded injury among children under 5 years of age found that 17% of burns were attributed to maternal depression (defined as clinical diagnosis of depression during pregnancy or in the first six months after delivery).^9^ Maternal depression was also found to be a risk factor for occurrences of poisoning, which comprised 33% of the injuries.^9^

A study conducted in England, in 2017, with data from the health services, investigated the relationship between duration of maternal depression or anxiety episodes and rates of poisoning, fractures, burns and serious injuries among children up to 5 years of age. Poisoning and burn rates increased as the duration of maternal depression and anxiety episodes increased.^10^

Depression is a common mental disorder that affects approximately 350 million people of all ages throughout the world, and women are most affected.^21^ Studies conducted on postpartum women show that the prevalence of depression may be as high as 40%.^22^ The arrival of a new baby generates physical, hormonal, mental and social changes that can place women in a vulnerable situation, which may directly reflect in their mental health and in the development of postpartum depression.^23^ Maternal depression is related to higher risks of behavioural problems among children, such as aggressiveness, impulsivity and hyperactivity.^24 25^ Children with behavioural problems are at a higher risk of injuries, as observed by Martins, in a systematic review of risk factors for injuries during childhood.^5^ Additionally, characteristics of depressive disorder itself like lethargy^26^ might cause mothers to pay less attention to security advice and child supervision, as well as to be less promptly responsive to unsafe situations.

In this study, the association between maternal depression and child injuries was more consistent among girls whose IRR values increased as the frequency of maternal depression symptoms increased. It is unclear whether girls would be more sensitive to maternal depression than boys or mothers of girls would report more easily the occurrence of any injury. There are reports that mothers protect girls more than they protect boys.^18^ A study conducted by Phelan *et al* 27 investigated maternal reports regarding the time spent taking care of their children during their first three years of life. A longer time was spent supervising girls than was spent on boys, and this supervision was considered by the mothers to be ‘intense’.^27^ In addition, studies conducted on animals showed that females had higher vulnerability to intrauterine stress.^28^ Other studies on animals indicated that female fetuses were more susceptible to anxiety, stress and depression during their adult stage if exposed to these factors during the prenatal period.^29^ Nonetheless, the only other study that conducted stratified analyses according to the child’s sex found a greater risk of injuries among boys than among girls of depressed mothers.^19^

Strengths and limitations of the current study should be recognised. Among the positive characteristics, the fact that it is a population-based study ensures the representativeness of the sample, allowing the results to be extrapolated to the same-age population in Pelotas and in settings with similar sociodemographic characteristics. The great majority of studies on childhood injuries were performed using samples from hospitals, emergency rooms and medical offices. Another positive aspect of the study was the low loss rate in all follow-ups, which may have helped to minimise selection bias. The follow-up period was also a positive factor because this study used data from the child’s birth and all reports of maternal depression were included from the time of pregnancy until the child reaches 24 months of age. Among the limitations, there was no information on the behaviour of the children until they were 4 years of age. Therefore, children’s behaviour could not be tested as a mediator variable between maternal depression and injuries. Nor were the situations that led to the injury investigated, which might have identified the person who was taking care of when the injury took place, and where it happened. In addition, no measure of severity was used to filter the injuries and no questions were asked regarding whether any medical care or first aid was required, thus eliciting that very minor lesions were recorded as an injury. However, the retrospective maternal recall to report on injuries in children over the previous two years would favour the report of more severe injuries. Also, two different approaches were used to build the composite variable maternal depression (self-reported maternal information from pregnancy and application of EPDS at 12-month and 24-month follow-ups). However, a previous analysis of the cohort showed a strong association between reported maternal depression in pregnancy and trajectories of depression (as assessed by EPDS) from 3 months to 6 years after the delivery.^25^ Also, because the information given was depended on the mother’s or caretaker’s perception of occurrences of injuries, it is possible that the injury rates among depressed mothers have been underestimated or overestimated.^30^

## Conclusion

Depression is a frequent health problem among women in reproductive ages, and the negative effect of maternal depression over the child health has been extensively reported in the literature.^26^ The current study adds consistency to the findings from recent researches^7–10^ showing that depressive symptoms in mothers are associated with the risk of injury among children and raises a new hypothesis that maternal depression may be especially risky for girls. Further studies specifically planned to address this objective as well as to explore the potential mechanism through which maternal depression leads to increased risk of injuries are needed. Recognition and management of maternal depression may help to promote maternal well-being and reduce child injuries.

**What is already known on the subject**

The association between maternal depression and child injuries has scarcely been investigated. Only four studies were found in the literature, which showed that postpartum maternal depression was associated with unintentional injury among children under 5 years of age.

**What this study adds**

In this population-based birth cohort, maternal depression during pregnancy and at 12 and 24 months post partum was associated with increased incidence of unintentional injuries among children aged 2–4 years, especially among girls.

10.1136/injuryprev-2017-042641.supp2 Abstract translation This web only file has been produced by the BMJ Publishing Group from an electronic file supplied by the author(s) and has not been edited for content.
