# Supplementary material for: Maternal depression is associated with injuries in children aged 2–4 years: the Pelotas 2004 Birth Cohort
Source: Inj Prev. 2018 Feb 26;25(3):222–7. doi: 10.1136/injuryprev-2017-042641 (PMC6582736; doi:10.1136/injuryprev-2017-042641)
Supplement: Supplementary file 1 [file injuryprev-2017-042641supp001.pdf]

Table 2A: Incidence of falls with respective 95% confidence interval (95% CI), according to the number of maternal depression episodes* (n = 3,533).

Maternal depression	Incidence of falls (95%CI)		
	**Boys**	**Girls**	**Total**
Never depressed	7.08 (6.60-7.59)	5.63 (5.16-6.15)	6.39 (6.05-6.76)
Depressed 1-2 times	7.09 (6.32-7.95)	7.45 (6.67-8.32)	7.27 (6.72-7.87)
Always depressed	8.14 (7.13-9.30)	7.55 (6.48-8.79)	7.86 (7.11-8.69)

* Edinburgh Postnatal Depression Scale (EPDS ≥ 13)

Table 2B: Incidence of cuts with respective 95% confidence interval (95% CI), according to the number of maternal depression episodes* (n = 3,533).

Maternal depression	Incidence of cuts (95%CI)		
	**Boys**	**Girls**	**Total**
Never depressed	0.51 (0.45-0.59)	0.42 (0.36-0.48)	0.47 (0.43-0.52)
Depressed 1-2 times	0.64 (0.54-0.76)	0.63 (0.53-0.74)	0.63 (0.56-0.71)
Always depressed	1.06 (0.87-1.28)	0.59 (0.45-0.76)	0.84 (0.71-0.98)

* Edinburgh Postnatal Depression Scale (EPDS ≥ 13)

Table 2C: Incidence of burns with respective 95% confidence interval (95% CI), according to the number of maternal depression episodes* (n = 3,533).

Maternal depression	Incidence of burns (95%CI)		
	**Boys**	**Girls**	**Total**
Never depressed	0.20 (0.17-0.22)	0.17 (0.14-0.20)	0.18 (0.17-0.20)
Depressed 1-2 times	0.23 (0.18-0.28)	0.19 (0.15-0.24)	0.21 (0.18-0.24)
Always depressed	0.25 (0.19-0.34)	0.22 (0.16-0.29)	0.24 (0.19-0.29)

* Edinburgh Postnatal Depression Scale (EPDS ≥ 13)

Table 3A: Incidence rate ratios of falls among children aged 24-48 months, according to exposure to maternal depression during pregnancy and at 12 and 24 months postpartum (EPDS ≥ 13). Pelotas 2004 Birth Cohort, RS, Brazil (n = 3,289)

Maternal depression	Falls (95% CI)					
	**Boys**		**Girls**		**Total**	
	**Crude**	**Adjusted[a]**	**Crude**	**Adjusted[b]**	**Crude**	**Adjusted[c]**
	p=0.09	p=0.17	p<0.001	p<0.001	p<0.001	p<0.001
**Never depressed**	1.0	1.0	1.0	1.0	1.0	1.0
**Depression 1-2 times**	1.00 (0.88-1.15)	1.02 (0.88-1.16)	1.32 (1.15-1.52)	1.32 (1.14-1.52)	1.14 (1.03-1.25)	1.16 (1.05-1.28)
**Always depressed**	1.15 (0.99-1.34)	1.12 (0.96-1.30)	1.34 (1.12-1.60)	1.36 (1.14-1.62)	1.23 (1.10-1.38)	1.22 (1.08-1.38)

Model adjusted for:
a: Mother's age, live with a partner, maternal smoking and child's skin color; **b:** Brazilian National Economic Index, mother's education level, mother's age, planned pregnancy, type of family and birth weigth; **c:** Brazilian National Economic Index, mother's education level, mother's age, maternal smoking, planned pregnancy, child's skin color and birth weight;

Table 3B: Incidence rate ratios of cuts among children aged 24-48 months, according to exposure to maternal depression during pregnancy and at 12 and 24 months postpartum (EPDS ≥ 13). Pelotas 2004 Birth Cohort, RS, Brazil (n = 3,496)

Maternal depression	Cuts (95% CI)					
	Boys		Girls		Total	
	Crude	Adjusted[a]	Crude	Adjusted[b]	Crude	Adjusted[c]
	p<0.001	p<0.001	p=0.04	p=0.12	p<0.001	p<0.001
**Never depressed**	1.0	1.0	1.0	1.0	1.0	1.0
**Depression 1-2 times**	1.24 (1.00-1.55)	1.11 (0.89-1.39)	1.50 (1.20-1.86)	1.34 (1.08-1.68)	1.35 (1.16-1.58)	1.21 (1.03-1.42)
**Always depressed**	2.05 (1.63-2.59)	1.63 (1.28-2.08)	1.41 (1.05-1.89)	1.20 (0.90-1.61)	1.78 (1.48-2.14)	1.46 (1.21-1.77)

Model adjusted for:

a: Brazilian National Economic Index, mother's education level, mother's age, maternal smoking, alcohol consumption during pregnancy and number of siblings in the household; **b:** Brazilian National Economic Index, mother's education level, mother's age, planned pregnancy, number of siblings in the household and type of family; **c:** Brazilian National Economic Index, mother's education level, mother's age, maternal smoking, alcohol consumption during pregnancy, planned pregnancy and number of siblings in the household;

Table 3C: Incidence rate ratios of burns among children aged 24-48 months, according to exposure to maternal depression during pregnancy and at 12 and 24 months postpartum (EPDS ≥ 13). Pelotas 2004 Birth Cohort, RS, Brazil (n = 3,291)

Maternal depression	Burns (95% CI)					
	Boys		Girls		Total	
	Crude	Adjusted[a]	Crude	Adjusted[b]	Crude	Adjusted[c]
	p=0.08	p=0.16	p=0.10	p=0.18	p=0.02	p=0.02
**Never depressed**	1.0	1.0	1.0	1.0	1.0	1.0
**Depression 1-2 times**	1.16 (0.90-1.51)	1.12 (0.86-1.45)	1.11 (0.84-1.47)	1.05 (0.79-1.41)	1.13 (0.93-1.37)	1.13 (0.92-1.38)
**Always depressed**	1.29 (0.95-1.77)	1.25 (0.90-1.72)	1.31 (0.94-1.81)	1.26 (0.90-1.77)	1.30 (1.04-1.63)	1.32 (1.04-1.68)

Model adjusted for:

a: Live with a partner, maternal smoking and alcohol consumption during pregnancy; **b:** Mother's education level, mother's age and alcohol consumption during pregnancy; **c:** Mother's education level, mother's age, alcohol consumption during pregnancy and child's skin color;
