# Supplementary material for: Maternal depression is associated with injuries in children aged 2–4 years: the Pelotas 2004 Birth Cohort
Source: Inj Prev. 2018 Feb 26;25(3):222–7. doi: 10.1136/injuryprev-2017-042641 (PMC6582736; doi:10.1136/injuryprev-2017-042641)
Supplement: Abstract translation [file injuryprev-2017-042641supp002.doc]

**ABSTRACT**

**Introduction:** Injuries during childhood, which mostly consist of falls, burns, drowning, poisonings and car crashes, are among the main causes of death among children and young adults in several countries. **Objectives:** To investigate the association between maternal depression and the incidence of injuries during childhood. **Methods:** In 2004, children who were born in the municipality of Pelotas, Brazil, were enrolled in a population-based birth cohort, with evaluations at birth and at 3, 12, 24 and 48 months of age. Maternal depression during pregnancy was evaluated at the time of delivery. At 12 and 24 months postpartum, the Edinburgh Postnatal Depression Scale (EPDS) was used. The injuries incidence rates at ages of 24-48 months and the crude and adjusted incidence rate ratios (IRRs) were calculated with 95% confidence interval (95% CI), through Poisson’s regression. **Results:** A total of 3,533 children were analyzed. The incidence of injuries was higher among children whose mothers presented depressive symptoms during pregnancy and at 12 and 24 months, compared with those whose mothers did not present any symptoms. In the adjusted analysis, the IRR among girls whose mothers presented depressive symptoms during pregnancy and EPDS ≥ 13 at 12 and 24 months was 1.31 (1.15-1.50); and, among boys, 1.18 (1.03-1.36). **Interpretation:** Maternal depression is associated with higher incidence of injuries between 24 and 48 months of age, in both sexes. **Funding:** Wellcome Trust, WHO, PRONEX, CNPq, Brazilian Ministry of Health, Children’s Pastorate.

**Keywords:** Cohort study, child, burn, fall, mental health
